# Microvesicles from Turmeric Extracts Contain Curcuminoids and Modulate Macrophage Polarization and Migration

**DOI:** 10.3390/pharmaceutics17121555

**Published:** 2025-12-03

**Authors:** Stefano Tacconi, Audrey Jalabert, Emmanuelle Berger, César Cotte, Elizabeth Errazuriz-Cerda, Valérie Bardot, Anne Leblanc, Lucile Berthomier, Michel Dubourdeaux, Sophie Rome

**Affiliations:** 1 CarMeN Laboratory (UMR INSERM 1060, INRA 1397), HCL-Lyon Sud, Lyon 1 University, 69152 Pierre-Bénite, France; 2 Laboratoire Ecologie Microbienne (LEM) (UMR CNRS 5557, INRAE 1418), VetAgroSup, University of Lyon, 69600 Villeurbanne, France; emmanuelle.danty@univ-lyon1.fr; 3 PiLeJe, 31–35 rue de La Fédération, 75015 Paris, Francea.leblanc@pileje.com (A.L.);; 4 CIQLE, Rockfeller Faculté de Médecine Lyon Est, Lyon 1 University, 69008 Lyon, France

**Keywords:** microvesicles, turmeric, curcumin, macrophages, immune response

## Abstract

**Background/Objectives:** Recent studies have revealed that plants produce lipid-derived microvesicles with potent anti-inflammatory properties. In turmeric (*Curcuma longa* L.), such microvesicles have been identified in rhizome juice and shown to exert beneficial effects in murine models of colitis. In this study, we investigated whether turmeric extracts commonly used in phytotherapy (30% ethanolic or aqueous extracts, and freeze-dried or spray-dried preparations) contain Curcuma-derived microvesicles (CuMVs), and we evaluated the influence of extraction processes on their aggregation and morphology. **Methods:** All extracts were processed using a standardized protocol involving differential centrifugation, filtration, and ultracentrifugation. CuMVs with sizes from 50 to 200 nm were detected in all pellets, but CuMVs from dehydrated extracts were markedly aggregated compared to those from liquid preparations. **Results:** The 30% ethanolic extract yielded the most polydisperse CuMVs and was therefore selected for functional immunomodulatory analyses on macrophages. Protein quantification indicated that 600 mL of 30% ethanolic extract contained approximately 60 µg of CuMVs which contained curcumin and its derivatives demethoxycurcumin (DMC), and bisdemethoxycurcumin (BDMC) identified by high-performance thin-layer chromatography (HPTLC). Green fluorescence in the form of small dots close to the nuclei was detected in recipient THP-1 macrophages, indicating the incorporation of CuMVs and therefore the transfer of the naturally fluorescent curcumin. CuMV treatment reduced ROS production, downregulated CD86, and upregulated CD163 expression. Furthermore, CuMVs increased the expression of IL-10 and TGF-β, as well as antibacterial cytokines (IL-1β, IL-6, and TNF-α), and enhanced RAW macrophage migration. Depletion of CuMVs from turmeric extracts markedly reduced their immunomodulatory effects. **Conclusions:** Collectively, these findings emphasize the importance of preserving CuMVs during the industrial processing of turmeric, as they play a crucial role in curcuminoid delivery and in mediating the immunomodulatory properties of turmeric extracts.

## 1. Introduction

Turmeric, an herbaceous plant, contains curcumin (C_21_H_20_O_6_), which is now recognized for its potential in the treatment of metabolic and inflammatory related diseases and for its antioxidant properties [1,2,3,4]. Unfortunately, curcumin is a fat-soluble compound, poorly soluble in water at neutral pH, and its bioavailability is limited. Consequently, it is recommended to consume curcumin with a high-fat meal to increase its absorption by mammalian cells, which is not recommended for subjects suffering from obesity. Various strategies were developed to offer solutions to increase curcumin bioavailability such as the addition of fatty substances to curcumin-like turmeric oil [5]; the use of piperine, which allows curcumin to cross the intestinal mucosa, or the use of rice flour, stearic acid, silica or magnesium stearate [6]; the use of methods to reduce the size of curcumin particles such as micelles, glucomannan fiber, glycerol fatty acids, polar lipids and solvents [7,8]; the use of natural complexes such as acacia gum, quillaia and sunflower oil for complete assimilation and optimal bioavailability [6]; and more recently the use of extracellular vesicles to complex curcumin to allow its passage through plasma membranes [9]. In that context, it was demonstrated that plants also contain lipid-derived microvesicles with anti-inflammatory [10], anti-cancer [11] or anti-diabetic properties [12]. These microvesicles have been isolated from juices extracted from fruits, roots or seeds [13]. Indeed, we have demonstrated that microvesicles from orange juice contained specific lipids able to restore the integrity of the intestinal barrier in high-fat diet obese mice [14]. In addition, it was demonstrated that ginger root-derived microvesicles protect against the development of liver-related diseases, such as alcohol-induced liver damage, recapitulating the hepatoprotective effect of ginger extract [15]. Ginger-derived vesicles have also the ability to regulate the innate immune response through their actions on NLRP3 inflammasome assembly and activation [16]. With regard to turmeric, recent studies have demonstrated the presence of microvesicles in the juice of its rhizome and their anti-inflammatory effects in mouse colitis models [17,18]. In [17], turmeric microvesicles administered orally could improve colitis and accelerate its resolution by regulating the expression of pro-inflammatory cytokines and inactivating the NF-κB pathway. In [18], turmeric microvesicles were shown to accumulate in the inflamed parts of the colon, to have anti-inflammatory activity, to regulate the gut microbiota and to promote the transformation of M1 to M2 macrophages. In published studies, microvesicles are generally extracted from the juice of the plant or fruit studied, as is the case for turmeric (e.g., [16]). In this study, we determined whether turmeric extracts such as those used in phytotherapy (liquid or dried extracts) contained microvesicles and assessed the impact of the extraction process on the level of aggregation and on the morphology of the extracted microvesicles. The objective was also to assess the effects of these microvesicles on the immune response by testing their ability to polarize macrophages.

## 2. Materials and Methods

### 2.1. Purification of Microvesicles from Turmeric Extracts

**Figure 1:**
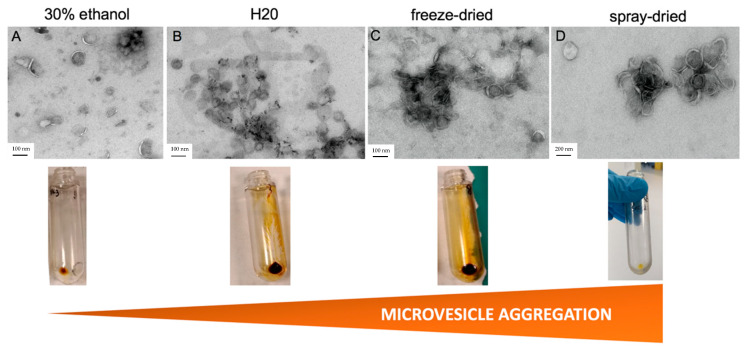
Pre-treatment of turmeric extracts affects the recovery and quality of CuMVs. Images from transmission electron microscopy of CuMVs isolated from liquid turmeric extracts in 30% ethanol (**A**), water (**B**), freeze-dried extracts (**C**) or spray-dried (**D**) extracts (bar = 200 nm for A and 100 nm for B-C-D). Aspects of the CuMV pellets from each extract after ultracentrifugation are shown below each image.

Frozen fresh turmeric roots (*Curcuma longa* L., harvested in Vietnam in January 2018) were ground and extracted with a mixture of alcohol and water (50:50; *v*/*v*). The dry matter yield of the extract was calculated with the dry matter of the plant and the extract is about 27%. The extract was concentrated under vacuum and resuspended in 30% alcohol or water. Two dehydration methods were also tested on the recovery of microvesicles: a freeze-drying method and a spray-drying method (Figure 1A). The resulting dehydrated compounds were resuspended in PBS. Then the protocol used to extract microvesicles was the same for all turmeric extracts and is presented in the Figure S1A. The liquid extract was centrifuged at 300× *g* to remove cell debris, fibers and various aggregates, and filtered across 4 layers of gauze bandage. The resulting eluate was centrifugated at 2000× *g* (30 min, 4 °C) and then at 10,000× *g* (60 min, 4 °C). After filtration at 0.45 µm (Nalgene^®^ bottle-top sterile filter units for large volumes or Eppendorf^®^ Membrane Filter (Hamburg, Germany) for small volumes), the eluate was ultracentrifuged (90 min, 100,000× *g*, 4 °C) and the supernatant was ultracentrifuged (90min, 100,000× *g*, 4 °C) (Beckman Coulter, Brea, CA, USA, Optima L-80 XP ultracentrifuge, type 50-2Ti rotor). The pellet was resuspended in PBS and re-pelleted in PBS after another ultracentrifugation (90 min, 100,000× *g*, 4 °C). Microvesicle proteins were quantified by Bradford assay. Size distributions in PBS pre-filtered at 0.1 µm were determined by Dynamic Light Scattering (DLS; n = 3 replicates, 20 °C, 17 runs of 60s/replicates, Dispersant RI = 1.332, Viscosity (cP)= 1.050) (Zetasizer, Malvern Panalytical, Malvern, UK).

### 2.2. Imaging of Turmeric Microvesicles with Transmission Electronic Microscopy (TEM)

Turmeric microvesicles resuspended in PBS were adsorbed on 200 mesh nickel grids coated with formvar, a polymer commonly used in electron microscopy. After washing once in filtered distilled water, suspensions were colored with 2% phosphotungstic acid for 2 min and examined using a JEM Jeol 1400 transmission electron microscope (Tokyo, Japan) equipped with an Orius 600 camera (West Palm Beach, FL, USA).

### 2.3. Macrophage Cell Culture and Treatments with CuMVs

THP-1 monocytes from human origin (ATCC TIB-202™) were grown at 37 °C with 5% CO_2_ in RPMI-1640 medium (Gibco™ Milieu RPMI 1640, Thermo Fisher Scientific, Waltham, MA, USA) modified to contain 2 mM L-glutamine, 10 mM HEPES, 1 mM sodium pyruvate, 4500 mg/L glucose, 1500 mg/L sodium bicarbonate and 100 IU/mL penicillin/streptomycin (Sigma-Aldrich, Saint Louis, MO, USA). Differentiation of THP-1 monocytes into M0 macrophages was induced by 100 ng/mL Phorbol 12-Myristate 13-Acetate (72 h). Then cells were incubated either with CuMVs (quantified with Bradford assay), or with 3% of total turmeric extract depleted or not from CuMVs by ultracentrifugation. Macrophage viability in response to increased concentrations of CuMVs was determined by crystal violet assay as reported in [19]. To visualize macrophage nuclear morphology in response to CuMVs, cells were washed twice in PBS and then fixed in 4% paraformaldehyde for 20 min. Fixed samples were stained with 4′,6-diamidino-2-phenylindole (DAPI) 1 mg/mL (20 min) and images were acquired with a LEICA microscope (40×). The number of fragmented/condensed nuclei was determined in seven randomly selected areas (0.2 mm^2^) from each experimental group.

To assess the anti-inflammatory activity of CuMVs, THP-1 macrophages were treated with 100 ng/mL lipopolysaccharide (LPS) and 10 ng/mL of interferon-γ (IFNγ) (pro-inflammatory stimulators) in presence or absence of CuMVs for 24 h.

### 2.4. Internalization of CuMVs in Recipient Macrophages

A total of 10^5^ THP-1 cells were differentiated onto 11 mm lysin-coated glass coverslip in 12-well plates. Macrophages were treated with CuMVs for three hours, washed with PBS and then fixed in 4% paraformaldehyde for 20 min. After three additional washes in PBS, samples were mounted on glass slides by using mounting media with DAPI (VECTASHIELD^®^ PLUS, H-2000, Newark, CA, USA) for nuclei staining. Images were captured with a fluorescence microscope (63×) (LEICA Imager Thunder, Wetzlar, Germany).

### 2.5. Quantification of Gene Expressions by qRT-PCR

At 24 h post-treatment total RNA was extracted from macrophages with TRIzol^®^ (Invitrogen, Carlsbad, CA, USA). A total of 1 mL of TRIzol^®^ in each well was used to lyse the cells. After 5 min at room temperature to allow complete lysis, 0.2 mL of chloroform was added, and the mixture was vortexed vigorously for 15 s and further incubated at room temperature for 2–3 min. The preparation was centrifugated at 12,000× *g* for 15 min at 4 °C. The aqueous phase was transferred into a new tube without disturbing the interphase. After the addition of 0.5 mL of isopropanol the mixture was gently agitated by inverting the tube, and incubated at room temperature for 10 min. The pellet of RNA was obtained after a centrifugation at 12,000× *g* for 30 min at 4 °C. The supernatant was discarded, and the RNA pellet was washed with 1 mL of 75% ethanol and resuspended in 20 μL RNase-free water. RNA (1 µg) was reverse transcribed (PrimeScript RT reagent kit (Takara, San Jose, CA, USA)). Real-time PCR was performed with the SYBR qPCR Premix Ex Taq (Tli RNaseH Plus) reagents (Takara), by using a Rotor-Gene Q (Qiagen, Venlo, The Netherlands).

List of primers (forward; reverse): CD86 (5′-AGGGAAGAGAGTGAACAGAC-3′; 5′-GTCGCATGAAGATGTCTTCG-3′), CD163 (5′-ACTCCAAAATCCAGGCAACA-3′; 5′-GCTTCACTTCAACACGTCCA-3′), IL-1β (5′-GTGTTCTCCATGTCCTTTGT-3′; 5′-CATATGGACCAGACATCAC-3′), IL-6 (5′-CAATCTGGATTCAATGAGGAGAC-3′; 5′-CTCTGGCTTGTTCCTCACTACTC-3′), TNFα (5′-AGCCCATGTTGTAGCAAACC-3′; 5′-GAGGTACAGGCCCTCTGATG-3′), IL-10 (5′-GATGCCTTCAGCAGAGTGAA-3′; 5′-GCAACCCAGGTA ACCCTTAAA-3′), TGF-β (5′-AGGGCTACCATGCCAACTTC-3′; 5′-GGTTATGCTGGTTGTACAGG-3′), TBP (5′-AGACCATTGCACTTCGTGCC; 5′-CCTGTGCACACCATTTTCCC).

### 2.6. Quantification of Macrophage ROS Production by FACS

Pre-treated THP-1 cells were trypsinized and fixed with formalin 10% (Sigma, Saint-Quentin Fallavier, France). After 20 min incubation at 4 °C, cells were centrifuged at 1000 rpm for 5 min (Eppendorf 5910R, Hamburg, Germany). The pellet was dissolved in 100 μL of dihydrorhodamine 123 (DHR, 5 μM) (Applied Bioprobes, Thermo Fisher Scientific, Waltham, MA, USA) in PBS with Triton 0.1X as described by the manufacturer. Cells were incubated with the DHR solution for 15 min at room temperature in the dark and analyzed by cell sorting (Novocyte flow cytometer, Acea Biosciences, San Diego, CA, USA). Data were collected and analyzed using the Software NovoExpress 1.2.5 (ACEA Biosciences, San Diego, CA, USA). Samples were prepared as three independent biological replicates and fluorescence intensities (FITC filter Excitation/Detection respectively 494/530 nm) were obtained for DHR-labeled cell population (DHR-H) by comparison to unlabeled control sample. Quantifications of ROS contents were normalized to cell counts.

### 2.7. Effect of CuMVs on RAW Macrophage Migration

RAW 264.7 cells (2 × 10^5^ cells/well) were seeded in 12-well culture plate for 24 h to reach the confluence. Scratched wound lines were created using a micropipette tip. After washing with culture medium RAW 264.7 were treated with 0.5 μg/mL of CuMVs for 24 h without FBS. The wounded area was visualized at time 0 h and 24 h using an inverted microscope (LEICA Imager Thunder) and the decrease in the gap area was quantified by ImageJ software (version 1.54i) as reported in [20].

### 2.8. Thin-Layer Chromatography Analyses of Turmeric Microvesicles

#### 2.8.1. Chemicals and Reagents

Solvents were all of chromatography grade. Methanol, ethanol, sulfuric acid, toluene and acetic acid were purchased from Carlo Erba (Val de Reuil, France). Magnesium chloride (≥98%) and methoxybenzaldehyde (≥99%, p-anisaldehyde, [4-methoxybenzaldehyde]) were obtained from Sigma-Aldrich-Fluka (Darmstadt, Germany). Curcumin 99% pure standard was obtained from Chromadex (Longmont, CO, USA).

#### 2.8.2. Sample Preparation

A total of 60 μg of CuMVs were stored for one week at −25 °C in 2 mL centrifuge tube until analysis. CuMVs were extracted with 2 mL of methanol by vortexing 1 min at room temperature, followed by ultrasonication (25 kHz) in a cold bath (kept at <4 °C) for 15 min. The suspension was centrifuged at 3000× *g* for 5 min. The yellow supernatant was collected and analyzed by HPTLC. For profile comparison, the raw turmeric ethanolic extract was diluted 10 times in ethanol–water 1:1 (*v*/*v*).

#### 2.8.3. High-Performance-Thin-Layer Chromatography

HPTLC analysis was performed on 10 cm^2^ HPTLC silica gel 60 F254 plates (Merck, Darmstadt, Germany) cut with a smartCut plate cutter (CAMAG, Muttenz, Switzerland). Samples were sprayed on as 8 mm bands (with a distance of 25 mm with the left edge and 8 mm to the bottom edge) using an Automatic TLC sampler 4 (ATS4, CAMAG). Sample volume was 150 µL for vesicle extracts and 2 µL for raw turmeric ethanolic extract. A total of 7 µL of a standard mixture of curcumin (0.15 mg/mL in methanol; 99% pure standard was also applied. In the Automated Development Chamber (ADC 2, CAMAG) after saturation for 20 min (with filter paper) and after activation at 33% relative humidity with a saturated solution of magnesium chloride, the plate was developed with a mixture of toluene–acetic acid 4:1, V/V up to a migration distance of 70.0 mm. The plate was dried for 10 min under a room-temperature air stream prior to derivatization. The chromatogram was piezoelectrically sprayed (blue nozzle, level 4, Derivatizer, CAMAG) with 2 mL of p-anisaldehyde reagent (mixture of 85 mL of methanol, 10 mL of acetic acid, 5 mL of sulfuric acid and 0.5 mL of p-anisaldehyde) and heated at 100 °C for 3 min. Documentation was performed at white light illumination (Vis) and UV 366 nm before and after derivatization (TLC visualizer, CAMAG).

### 2.9. Statistical Analysis

Results are presented as mean values ± standard deviation (SD). Data distribution, group comparisons and graphical representations were analyzed using GraphPad Prism 9 (GraphPad Software Inc., San Diego, CA, USA). A p-value of less than 0.05 was considered indicative of statistical significance. The specific statistical tests applied in each case are detailed in the corresponding figure legends.

## 3. Results

### 3.1. Pre-Treatments of Turmeric Extracts Affect the Recovery and the Morphology of Turmeric Microvesicles (CuMVs)

To identify the best process for recovering the maximum number of microvesicles with good morphologies, different turmeric extracts, resuspended in 30% alcohol, in water, or dehydrated were tested. Two liquid extracts and two dehydrated extracts, the latter resuspended in PBS, were tested (Figure S1A). Purified CuMVs were resuspended in PBS. All aqueous turmeric extracts were treated in the same way following a classical protocol based on differential centrifugations, followed by an ultracentrifugation step (Figure S1B). As the composition of turmeric CuMVs was unknown, commercial kits that could have selected microvesicles based on size only (e.g., size exclusion chromatography) or on their composition (e.g., density gradients) or on both [14] were not used. CuMVs were visualized by TEM, using a protocol developed for microvesicles from human origin [21] (Figure 1A–D). The results showed the presence of CuMVs from various sizes in all pellets and the absence of impurities. However, CuMVs extracted from dehydrated extracts were strongly aggregated compared to microvesicles from liquid preparations (Figure 1A,B vs. Figure 1C,D).

**Figure 2:**
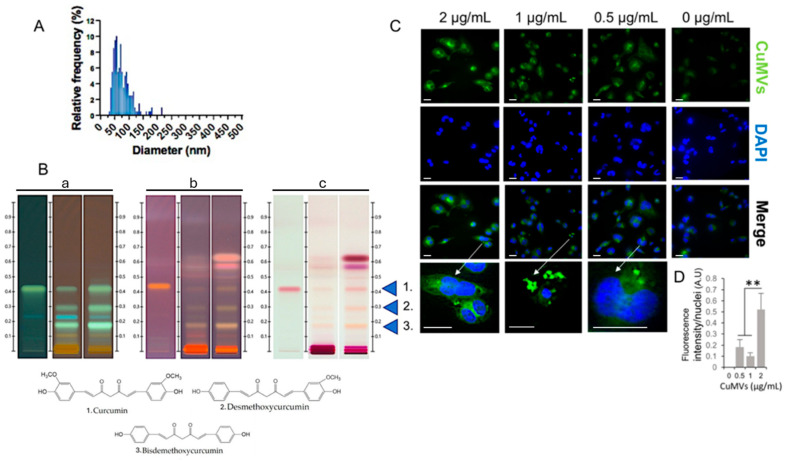
CuMVs containing curcumin transferred into recipient macrophages. (**A**) Analysis of CuMV size distribution from TEM images (distribution confirmed by Dynamic Light Scattering in S2); (**B**) Detection of curcumin and its derivatives in CuMVs (60 μg) by High-Performance-Thin-Layer Chromatography (**a** = before derivatization at 366 nm; **b** = after derivatization at 366 nm; **c** = after derivatization visualized with white light, 1 = Curcumin; 2 = Demethoxycurcumin; 3 = Bisdemethoxycurcumin). (**C**) Representative images of CuMVs internalized into macrophages (scale bar 50 μm). Arrows indicate which cell has been magnified. (**D**) Quantification of internalized fluorescent CuMVs in THP-1 macrophages by detecting fluorescent curcumin associated to CuMVs (excitation: 496 nm, detection: 517 nm), ** = *p* < 0.05 (one-way ANOVA).

As CuMVs in the 30% alcoholic preparation were the most polydispersed, this extract was selected for functional studies. Based on protein quantification with the Bradford assay, we obtained a quantity of 60 µg of microvesicles in 600 mL of 30% alcoholic extract. CuMVs had sizes between 50 nm and 200 nm (Figure 2A and Figure S1).

### 3.2. CuMVs Contain Curcumin and Are Internalized into Recipient Macrophages

**Figure 3:**
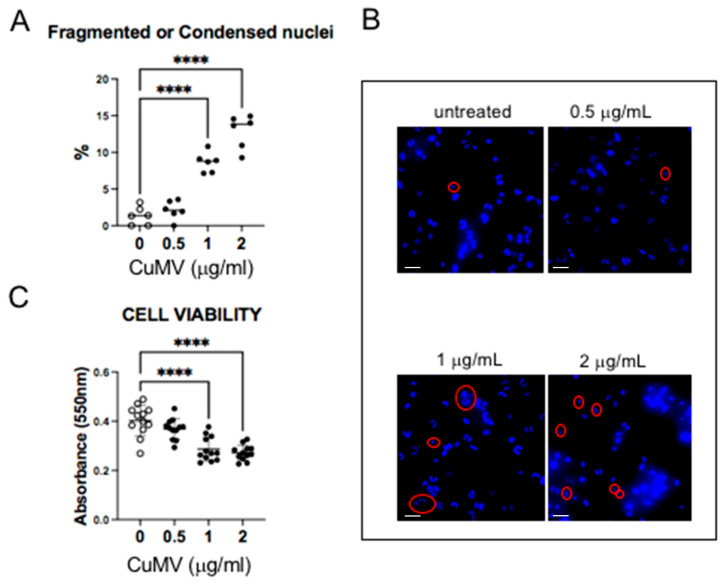
(**A**,**B**) Representative images of THP-1 with fragmented or condensed nuclei in response to CuMVs and quantifications. Scale bar: 50 μm. Red circles indicate fragmented or condensed nuclei. (**C**) Analysis of THP-1 cell viability in response to CuMVs. **** = *p* < 0.001 vs. control (one-way ANOVA).

As all pellets of CuMVs had a yellow-orange color that was not removed after washes in PBS (Figure 1), we suspected that CuMVs could contain non-soluble curcumin. This was confirmed by the HPTLC analyses, which showed that CuMVs contained curcumin and its derivatives demethoxycurcumin (DMC) and bisdemethoxycurcumin (BDMC) (Figure 2B). Then we treated THP-1 macrophages with different concentrations of CuMVs. Green fluorescence in the form of small dots close to the nuclei was detected indicating the incorporation of CuMVs and therefore the transfer of curcumin inside recipient macrophages (Figure 2C,D). Then we tested the viability of the recipient macrophages in response to different concentrations of CuMVs by determining the percentage of recipient cells with condensed or fragmented DNA using increased concentrations of CuMVs. Above 0.5 µg/mL, CuMVs induced DNA alterations in recipient macrophages (Figure 3A,B) and affected their viability (Figure 3C). This was confirmed at the RNA level as after 24 h of treatment, the quantity of RNA extracted was significantly reduced above 1 µg/mL of CuMVs (Figure S3A). Therefore, we used a non-toxic concentration of 0.5 µg/mL of CuMVs to test the immunomodulatory properties of CuMVs.

### 3.3. CuMVs Modulate Macrophage Migration and Polarization

**Figure 4:**
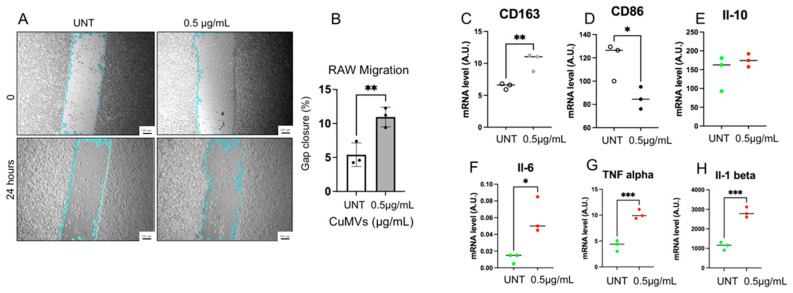
CuMVs modulate macrophage migration and polarization. (**A**) Representative images showing the wound healing assay to study macrophage migration (scale bar= 100 μm). (**B**) Percentage of gap closure 48 h after incubation with 0.5 µg of CuMVs. (**C**–**H**) RT-PCR quantification of markers of macrophage polarization and cytokines by qRT-PCR in THP-1 macrophages treated with CuMVs (data normalized to TBP, Arbitrary Units). * = *p* < 0.05, ** = *p* < 0.01, *** = *p* < 0.001, vs. control (Student’s *t*-test).

As macrophage migration is a crucial process in the immune response to reach sites of infection or inflammation, we first determined whether CuMVs were able to affect macrophage migration. RAW macrophages were grown until confluence, and a scratch at the center of monolayer was made. The closure of the gap was determined after 24 h. As shown in Figure 4A,B, CuMVs increased macrophage migration, suggesting that CuMVs could participate in the immune-modulatory effects of turmeric. To validate this hypothesis, THP-1 M0 macrophages were cultured in the presence of CuMVs and we quantified the expression of M1 and M2 markers by qRT-PCR. THP-1 M0 macrophages are a mixed population that expresses both CD86 and CD163 markers specific to M1 or M2 macrophages, respectively (Figure 4C,D). But after the treatment with CuMVs, macrophages expressed a higher level of CD163 and had decreased expression of CD86 (Figure 4C,D). CuMV-treated macrophages had no modifications of IL-10 expression (Figure 4E) but higher expressions of IL-6 (Figure 4F), TNFα (Figure 4G) and IL-1β (Figure 4H).

In order to test the biological activity of exogenous microvesicles, it is recommended to use a microvesicle-free FBS that is ultracentrifuged before use [22]. As macrophages are very sensitive to culture conditions, it was important to validate that these results were independent of the FBS pre-treatment. Indeed, it was demonstrated that macrophages grown in microvesicle-free FBS had altered cytokine responses [23]. The results showed that CD86 expression was not affected by the presence of microvesicles contained in bovine serum and that the CD86 decrease in the presence of CuMVs observed in Figure 4D was reproduced even without serum (S3B). On the other hand, CD163 expression decreased in a medium depleted in serum microvesicles (S3C). However, as CD163 increased in the presence of CuMVs, the effect shown in Figure 4C is due to the presence of CuMVs.

### 3.4. CuMVs Modulated ROS Production in Recipient Macrophages

**Figure 5:**
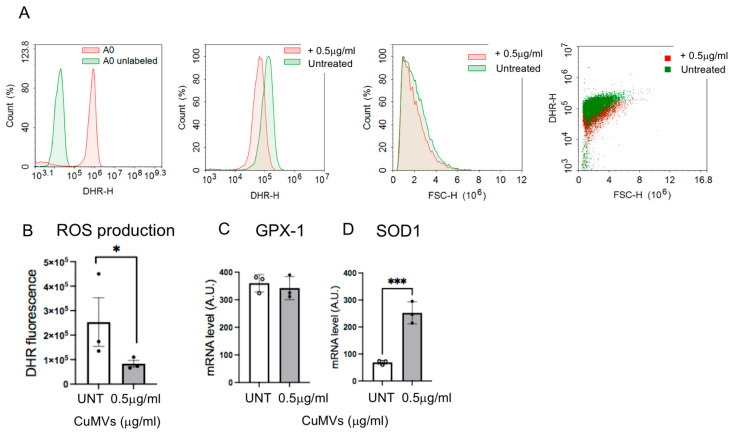
Quantification of ROS production by FACS analyses (representative analyses in (**A**)) in THP-1 treated with CuMVs. (**B**) Data are normalized to the number of macrophages. (**C**,**D**) Quantification of SOD1 and GPX1 mRNA by qRT-PCR, in THP-1 macrophages treated with CuMVs (data normalized to TBP and expressed as Arbitrary Units). * = *p* < 0.05 and *** = *p* < 0.001, vs. control (Student’s *t*-test).

Based on a previous study showing that ROS levels in macrophages control their differentiation and polarization [24], we investigated whether CuMVs could modulate ROS production in recipient macrophages. Therefore, we quantified ROS production in response to CuMVs by FACS (Figure 5A,B). The results showed that CuMVs induced a decrease in ROS production in recipient macrophages (Figure 5B). The expression of Glutathione peroxidase-1 (GPX-1) was not affected (Figure 5C) but SOD1 expression increased (Figure 5D).

**Figure 6:**
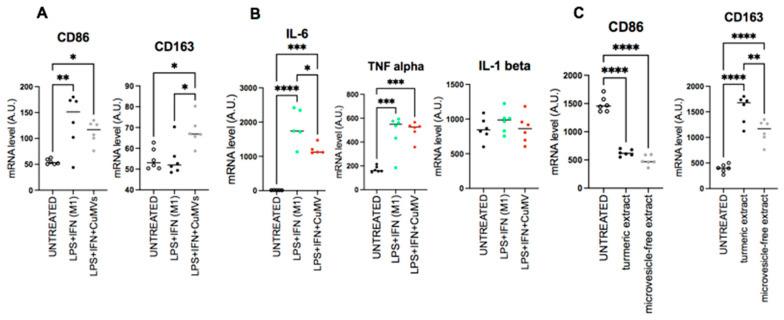
CuMVs modulate markers of polarization in macrophages. (**A**–**C**) mRNA quantification of genes involved in macrophage polarization or (**B**) coding for cytokines. (data normalized to TBP, Arbitrary Units). * = *p* < 0.05; ** = *p* < 0.01; *** = *p* < 0.001; **** = *p* < 0.0001 (one-way ANOVA).

Then we determined the anti-inflammatory properties of CuMVs on macrophages polarized into M1 with LPS (Figure 6A). CuMVs induced a significant increase in CD163 expression and a small non-significant decrease in CD86 reproducing the effect of CuMVs observed in Figure 4C,D. This shift in macrophage phenotype was associated with a decrease in IL-6 expression but not in TNFα and IL-1β (Figure 6B).

Finally, we validated that CuMVs participated in the biological activity of turmeric extract. To do this, we compared the polarization of macrophages incubated in the presence of the complete turmeric extract with the same extract without CuMVs removed by ultracentrifugation. The complete turmeric extract induced an increase in CD163 expression and a decrease in CD86 expression (Figure 6C) reproducing the results obtained with CuMVs (Figure 3). When CuMVs were removed from the turmeric extract by ultracentrifugation, treated macrophages had no modification of CD86 expression but a significant decrease in CD163 mRNA levels compared to the turmeric extract condition (Figure 5C).

## 4. Discussion

In this study, we confirm the presence of microvesicles (CuMVs) in extracts from turmeric roots with anti-inflammatory properties [16,18]. We also demonstrated that removal of CuMVs from turmeric extracts strongly reduces the anti-inflammatory properties of turmeric and, for the first time, that CuMVs contain curcumin and its bioactive derivatives, which can be transferred into recipient macrophages, participating in the effect of CuMVs on macrophage migration and polarization.

Our results show that curcumin is naturally associated with microvesicles in turmeric extracts, which likely enhances curcumin entry into recipient cells because of the lipidic nature of CuMVs. These data indicate that it is now important to reconsider the curcumin extraction processes from turmeric to keep intact as many microvesicles as possible. Turmeric is consumed either dehydrated or as a liquid, which contains liposomes complexed with curcumin. Manufacturers offer turmeric extracts that are made from ground turmeric roots enriched in curcuminoids. These extracts must be taken with foods containing fats (e.g., coconut oil or milk) to be absorbed. CuMVs were found in the dry extract, but our data show an impact of dehydration on the quantity of CuMVs recovered. Dehydration of turmeric extracts also affects CuMV morphology. Nonetheless, the microvesicles still contain curcumin. In addition, we showed that the solvent used to resuspend concentrated turmeric extracts (H_2_O vs. alcohol) has an impact on CuMV aggregation. Aggregation makes it difficult to reproduce the extraction in terms of CuMV concentrations. It was beyond the scope of this study to test all solvent preparations, but it would be interesting to determine the consequences on macrophage polarization. Indeed, in a previous study on orange juice, we demonstrated that dehydration or filtration removed virtually all the microvesicles contained in orange juice, and thus their important biological effect on the permeability and lipid metabolism of the intestinal barrier [14].

In this study, we have focused on the concentrated turmeric extract resuspended in 30% alcohol, which showed nice and poorly aggregated CuMV morphology, as estimated by electron microscopy. Among the possible CuMV recipient cells in the digestive tract, we observed that the macrophages integrated CuMVs but that high CuMV concentrations induced apoptosis.

Naive macrophages treated either with low concentrations of CuMVs or with turmeric extract had higher expression of CD163, a maker of M2 polarization. Interestingly, removal of CuMVs from the turmeric extract before macrophage treatment prevents this increase. These data are in line with the role of CuMVs in the anti-inflammatory action of turmeric, previously demonstrated in vivo on a model of mice colitis [17,18]. In addition, when M1 macrophages were treated with CuMVs, they displayed an increase in CD163 expression suggesting that CuMVs could also be used to reduce inflammation.

CD163^+^ macrophages play an important role in wound healing and tissue repair. However, it has been shown that this immunosuppressive function promotes also tumor growth on a long-term basis [25]. Here, we show that in presence of CuMVs, the CD163^+^ macrophages retained the property of expressing both anti-tumor cytokines IL-10 and TGFβ. We explain this result by the fact that CuMVs can modulate ROS production and the expression of superoxide dismutase (SOD1) in recipient macrophages, thus preventing the transition of M2 macrophages into TAMs (tumor-associated macrophages) [26]. Interestingly, CD163^+^ macrophages exposed to CuMVs also expressed pro-inflammatory and antibacterial cytokines (IL-1β, IL-6 and TNF-α), which are characteristic of M1 macrophages. These findings might suggest that CuMV-treated macrophages do not conform strictly to the classical M1/M2 polarization model. Instead, they acquire a M2b/M2d phenotype, combining anti-inflammatory and antimicrobial properties [27].

The other important discovery is the presence of curcumin in CuMVs, which can be transferred into recipient cells. Taking advantage of the curcumin autofluorescence, we were able to follow its incorporation into recipient THP-1 cells. It has been known for many years that the low bioavailability of curcumin is a limiting factor regarding the pharmacological effect because curcumin is not absorbed by cells and must be complexed with lipids in order to cross cell membrane. Our results show that curcumin is naturally associated with microvesicles inside turmeric extracts, which are therefore participating in curcumin entry into recipient cells, as we demonstrated that CuMVs are incorporated into macrophages. This result reinforces the idea it is now important to reconsider the curcumin extraction process from turmeric to keep intact as many microvesicles as possible because they contribute to the “health” effects of the turmeric extracts and help to pass curcuminoids through cell membrane. In addition to curcumin, CuMVs also contain curcumin derivatives such as demethoxycurcumin (DMC) and bisdemethoxycurcumin (BDMC). Together with curcumin, DMC and BDMC are strong anti-cancerous agents [28] and have also anti-inflammatory properties [29]. Therefore, DMC and BDMC in CuMVs are likely to participate in the effects of CuMVs observed on macrophage polarization.

## 5. Conclusions

In this study, we show for the first time that microvesicles previously reported in turmeric extracts by other authors [18] can be preserved after ethanolic extraction and processing (spray-drying and dehydration). Furthermore, the in vitro results show that the immuno-modulatory activity of microvesicles is also maintained after ethanolic extraction. This brings new insights in understanding the mode of action of plant extracts. A low concentration of microvesicles could explain the health effects observed in traditional medicine for certain extracts of native turmeric with low curcuminoid content (i.e., non-enriched extracts). In the future, it would be interesting to assess how different extraction processes impact the content and quality of microvesicles with the aim of obtaining optimized extracts in terms of efficacy and but also safety. Indeed, while curcumin and its derivatives are generally regarded as safe at dietary levels, several clinical and preclinical studies have reported dose-dependent hepatotoxicity associated with high intake of purified or highly concentrated curcumin supplements [30]. This toxicity is thought to result from the formation of reactive metabolites and the potential for oxidative stress in hepatocytes. Therefore, preserving the natural microvesicular form of curcumin, as found naturally in turmeric extracts, could represent a safer and more physiologically balanced delivery system that maintains bioactivity while reducing the risk of liver toxicity. This has to be tested in vivo.

The science of plant microvesicles represents an exciting opportunity for the field of herbal medicine, but it is still in its early stages, and many challenges, such as the requirement for standard, reproducible and inexpensive processing techniques, need to be overcome before it can be scaled up.

## Data Availability

The original contributions presented in this study are included in the article/Supplementary Material. Further inquiries can be directed to the corresponding authors.

## Supplementary Materials

The following supporting information can be downloaded at https://www.mdpi.com/article/10.3390/pharmaceutics17121555/s1. Figure S1. Description of the pre-treatments applied on the turmeric extracts and summary of the experiments (A). Protocol used to extract microvesicles from all pre-treated extracts described in (B). Figure S2. Size distribution of CuMVs measured by dynamic light scattering (DLS). Figure S3. (A) Quantity of RNA extracted from TPH-1 macrophages after a treatment for 24 h with different concentrations of CuMVs. (B) Comparison of CD163 and CD86 mRNA levels from macrophages grown in different media. * = *p* < 0.05 vs. control (Student’s *t*-test).
