# Supplementary material for: Microvesicles from Turmeric Extracts Contain Curcuminoids and Modulate Macrophage Polarization and Migration
Source: Pharmaceutics. 2025 Dec 3;17(12):1555. doi: 10.3390/pharmaceutics17121555 (PMC12736489; doi:10.3390/pharmaceutics17121555)
Supplement: Supplementary file 1 [file pharmaceutics-17-01555-s001.zip › pharmaceutics-3955215-supplementary.pdf]

Figure S1

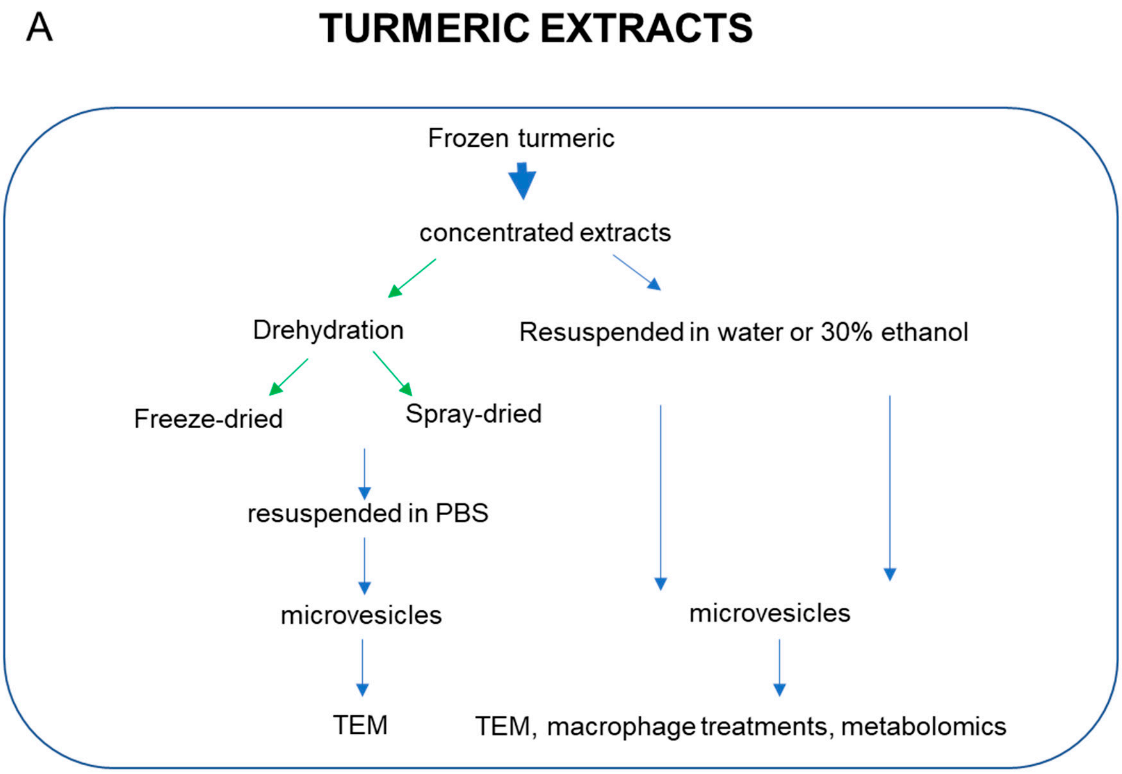
A
TURMERIC EXTRACTS
Frozen turmeric
concentrated extracts
Drehydration
Resuspended in water or 30% ethanol
Freeze-dried
Spray-dried
resuspended in PBS
microvesicles
microvesicles
TEM
TEM, macrophage treatments, metabolomics

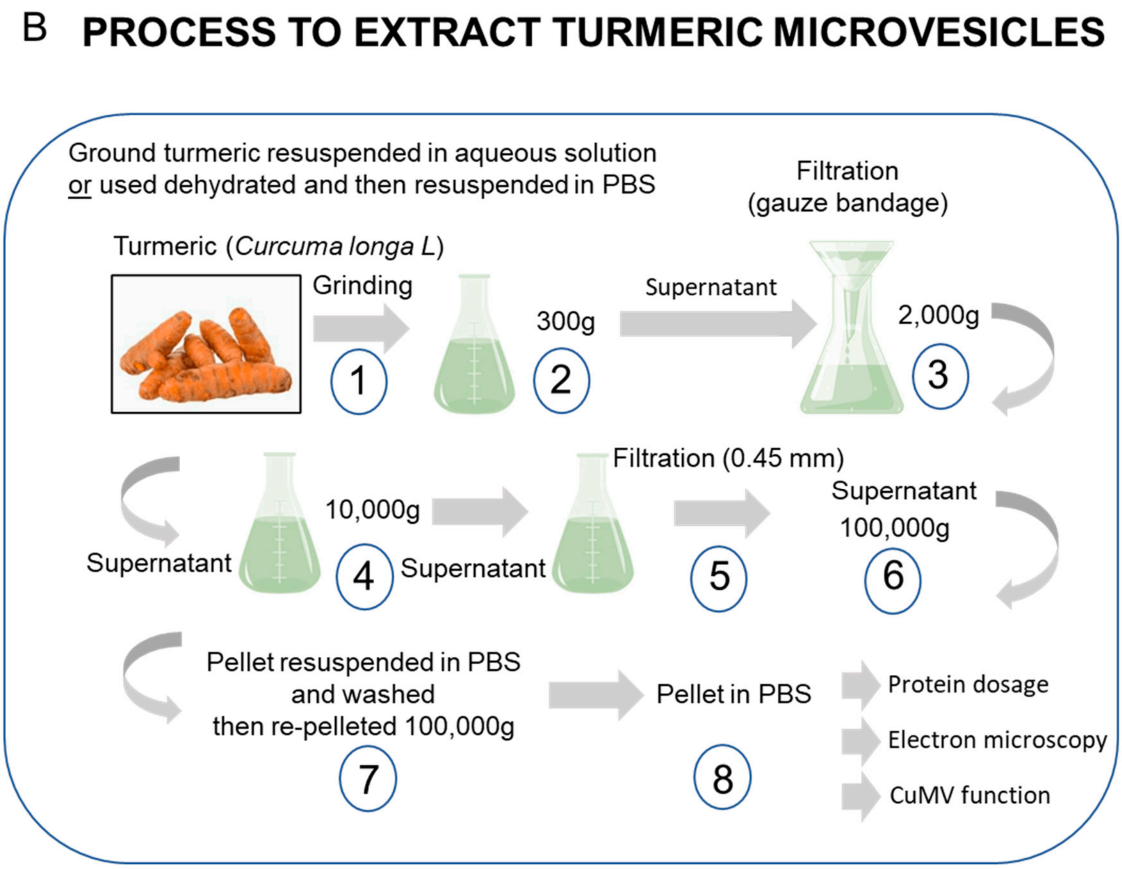
B
PROCESS TO EXTRACT TURMERIC MICROVESICLES
Ground turmeric resuspended in aqueous solution
or used dehydrated and then resuspended in PBS
Filtration
(gauze bandage)
Turmeric (*Curcuma longa L*)
Grinding
1
300g
2
Supernatant
2,000g
3
Supernatant
10,000g
4
Supernatant
Filtration (0.45 mm)
5
Supernatant
100,000g
6
Pellet resuspended in PBS
and washed
then re-pelleted 100,000g
7
Pellet in PBS
8
Protein dosage
Electron microscopy
CuMV function

Figure S2

Size distribution of 3 independent preparations of CuMVs determined by Dynamic Light Scattering

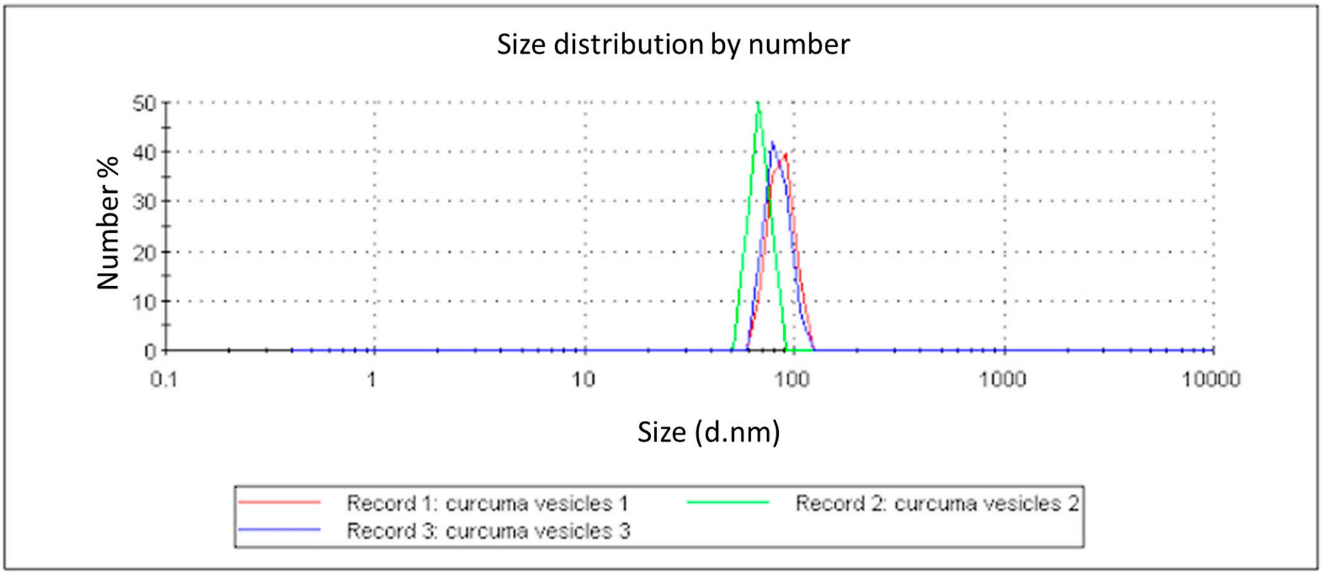

Figure S3

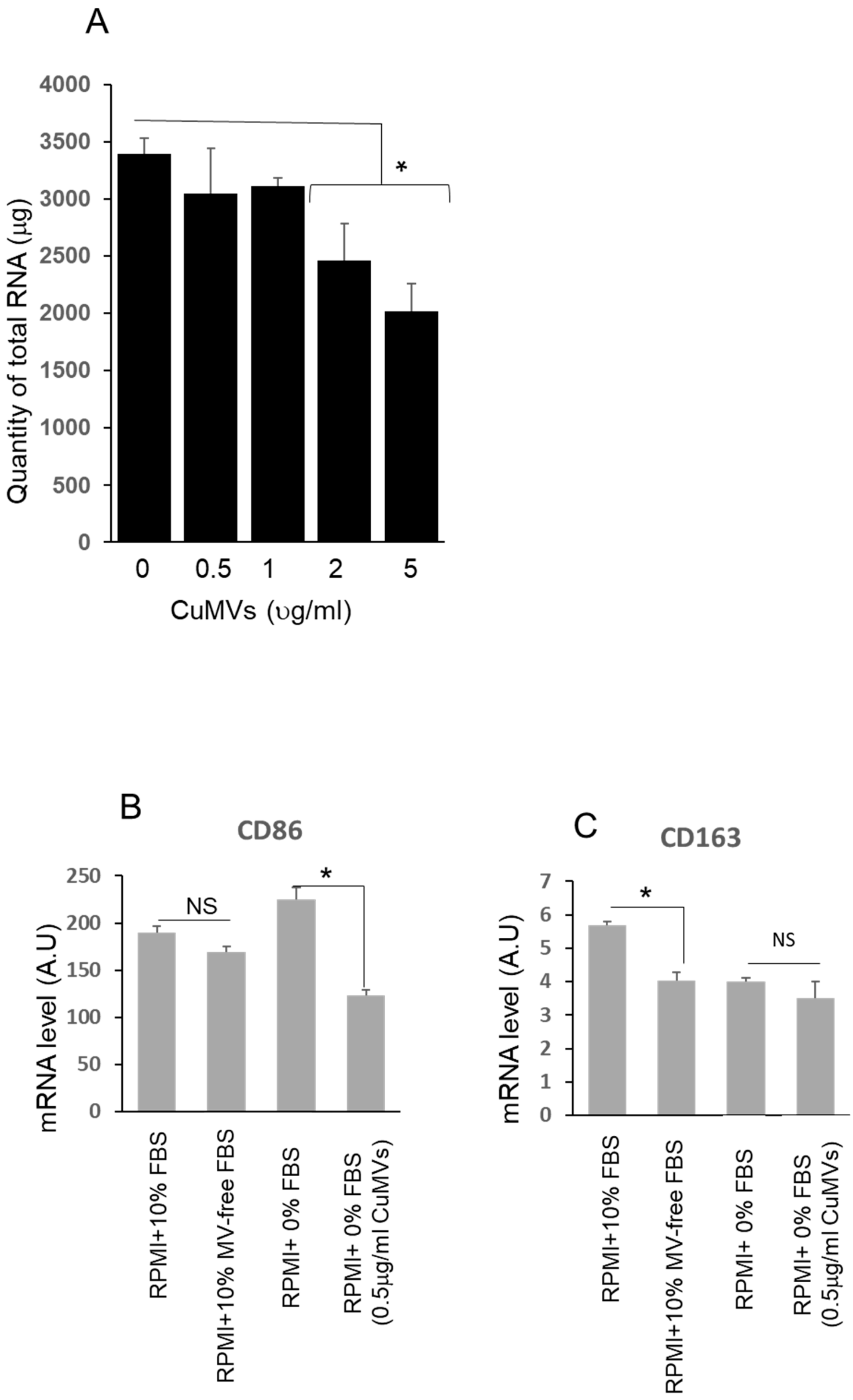
A
4000
3500
3000
2500
2000
1500
1000
500
0
Quantity of total RNA (µg)
*
0
0.5
1
2
5
CuMVs (υg/ml)
B
CD86
250
200
150
100
50
0
mRNA level (A.U)
NS
*
RPMI+10% FBS
RPMI+10% MV-free FBS
RPMI+ 0% FBS
RPMI+ 0% FBS (0.5µg/ml CuMVs)
C
CD163
7
6
5
4
3
2
1
0
mRNA level (A.U)
*
NS
RPMI+10% FBS
RPMI+10% MV-free FBS
RPMI+ 0% FBS
RPMI+ 0% FBS (0.5µg/ml CuMVs)
